# “Sex Out of Boredom”: Key Stakeholders’ Perspectives on Teen Pregnancy Prevention in Emerging Immigrant Latino Rural Communities

**DOI:** 10.1007/s13178-024-00967-8

**Published:** 2024-05-11

**Authors:** Romina L. Barral, J. Dennis Fortenberry, Astrid Guerrero Avitia, Mariana Ramirez, Abbey R. Masonbrink, Claire D. Brindis

**Affiliations:** 1 Division of Adolescent Medicine, Children‘s Mercy Kansas City, University of Missouri-Kansas City School of Medicine, 3101 Broadway Blvd, Kansas City, MO 64111 USA; 2 School of Medicine, University of Kansas Medical Center, Kansas City, KS USA; 3 Division of Adolescent Medicine, Indiana University School of Medicine, Indianapolis, IN USA; 4 Universidad Autónoma del Estado de Morelos, Morelos, Mexico; 5 Division of Population Health, Medical Center, University of Kansas, Kansas City, KS USA; 6 Department of Pediatrics, Children‘s Mercy Kansas City, University of Missouri-Kansas City School of Medicine, Kansas City, MO USA; 7 Department of Pediatrics, Adolescent and Young Adult Health National Resource Center, Philip R. Lee Institute for Health Policy Studies (IHPS), University of California San Francisco, San Francisco, CA USA

**Keywords:** Key stakeholders, Teen pregnancy, Immigrant, Latino, Rural, Adolescents and young adults

## Abstract

**Introduction:**

Teen pregnancy (TP) rates are 1.5 times higher among Latina youth than the United States national average and one-third times higher in rural counties. The Socio-Ecological framework recognizes the myriad of issues that impact TP, including four bidirectional levels of influence on teenagers’ behaviors: macro, community, institutional, and interpersonal levels. We aim to fill critical knowledge gaps regarding the influence of key community stakeholders living in rural, Latino-majority communities shaping Latino/a, immigrant adolescents’ TP-related environments.

**Methods:**

A purposive sample of 48 key stakeholders was drawn from three rural counties (Finney, Ford, and Seward) in southwestern Kansas from 2016 to 2017; participants completed a brief demographic survey and a semi-structured qualitative interview. Qualitative data analysis followed grounded theory within a Socio-Ecological framework, and we used descriptive statistics to analyze survey data.

**Results:**

Respondents (*N* = 48) included 5 public health department staff, 8 community health workers, 8 healthcare workers, 9 community members, and 18 high school/college administrators. The mean age was 43 years (SD = 15.5) and 50% self-identified as Latino/a. Recommendations included developing TP prevention education programs for parents, utilizing ongoing events and familiar venues, and keeping content consistent with local culture and norms.

**Conclusions:**

Key stakeholders’ perceptions regarding TP are often unaccounted for but play a role in shaping youth’s decision-making environments.

**Policy Implications:**

This information could inform the development of culturally specific TP prevention interventions, especially considering the controversial politics centered on immigration to the U.S. and its negative impact on the overall health of Immigrant Latinos living in the U.S.

## Introduction

Teenage pregnancy (TP), of which 75% is unintended, can lead to adverse health, educational, and economic outcomes for both the mother and the child, costing over $9.4 billion annually in public care (National Campaign to Prevent Teen and Unplanned Pregnancy, 2013; CDC, 2016). TP rates have declined in the United States (U.S.) in the last several decades. However, rates not only continue to surpass the rates of other developed countries (Boonstra, 2002), but disparities continue to persist. The latest data on teen birth rates among Latina teens in the U.S. indicate that they are 1.5 times higher than their white counterparts (24 vs 10 births per 1000 girls 15–19 years old in 2020) (Martin et al., 2019). In rural counties, Latina teens have birth rates almost 2 times higher as compared to White rural teens (47 vs 26.8 births per 1000 15–19-year-olds in 2015, the latest information available) (Martin et al., 2019; Ng & Kaye, 2015; Hamilton, 2016).

Access to reproductive healthcare (RHC) in rural America is particularly challenging due to the shortage of family planning specialists working in these areas, lack of confidential services, and remote location of services coupled with limited transportation (Ng & Kaye, 2015). Additionally, traditional community attitudes and beliefs often contribute to limited access to RHC in rural areas (Ng & Kaye, 2015). 

Compounding these historical challenges, rural America has continued to develop industries, such as meatpacking and agriculture, that have attracted a growing number of immigrant workers over the past 3 decades (Housing Assistance Council, 2012). Many of these workers migrate directly from countries in Latin America, often bypassing established migration routes, and result in a marked rural Latino population growth, shifting the demographic landscape of the U.S. (Turner et al., 2016).

These groups face special healthcare needs and access obstacles that are somewhat different from those of established Latino communities, including indigenous languages and cultural barriers. Available job opportunities often require adults working long hours, weekend shifts, and hazardous job-related tasks, often coupled with few healthcare benefits (Topmiller et al., 2017). Additionally, families face adjustment difficulties in unfamiliar settings, coupled with lacking experience navigating new, difficult, and complex systems of healthcare under the shadow of increasingly restrictive immigration policies (Topmiller et al., 2017).

### Socio-Ecological Framework to Study Teenage Pregnancy Prevention

**Fig. 1:**
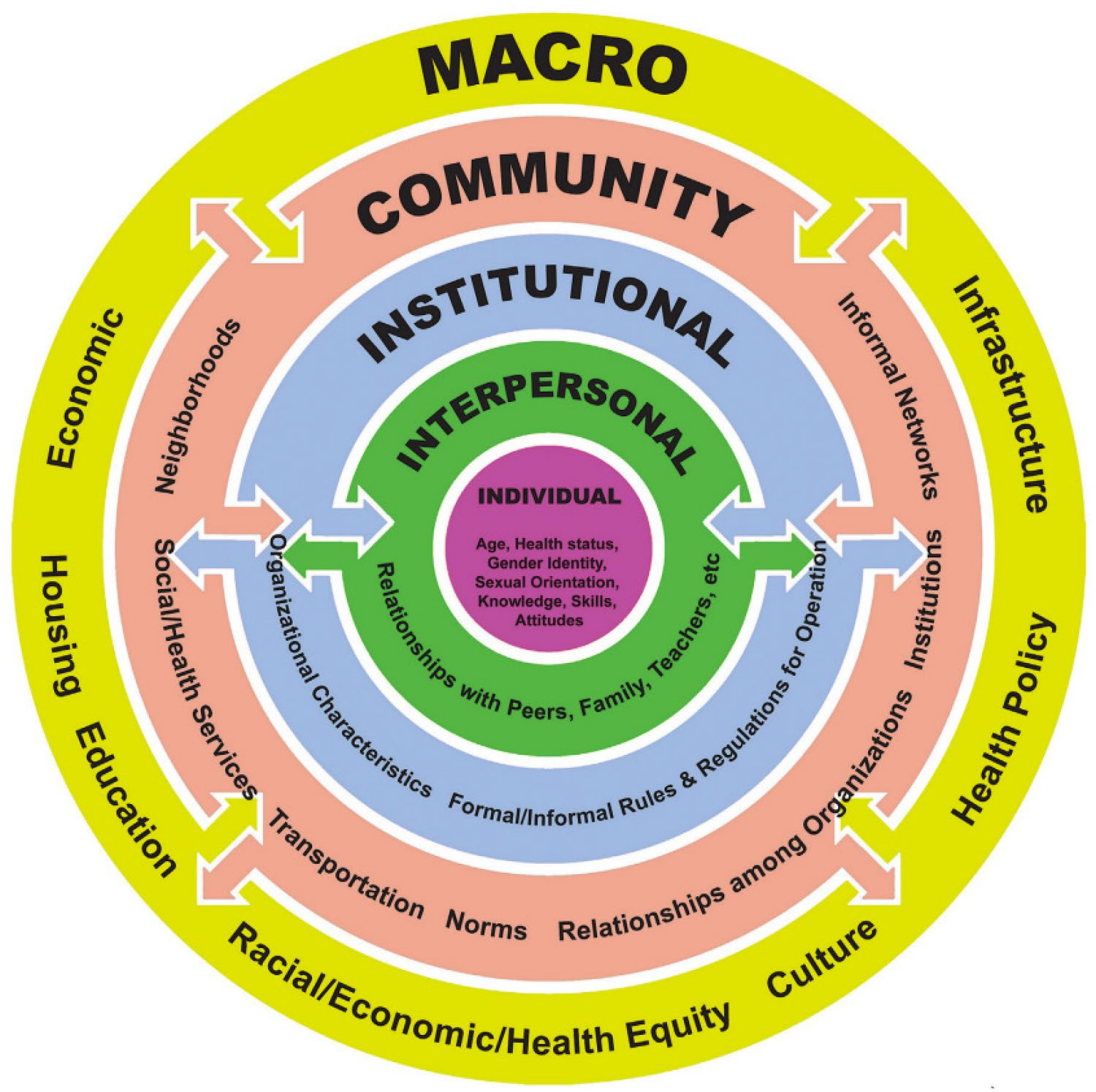
Socio-Ecological model (Tebb & Brindis, 2022)

The Socio-Ecological model considers four bidirectional levels of influence on teenagers’ behaviors: macro, community, institutional, and interpersonal levels (see Fig. 1) (Tebb & Brindis, 2022; CDC, 2022; Bronfenbrenner, 1992). Increasingly, the field of TP has adopted a Socio-Ecological framework to consider the variety of external and contextual issues that shape the environments in which adolescents make their individual decisions (Tebb & Brindis, 2022; CDC, 2022). This includes their ability to access limited TP prevention resources, including sexual and reproductive health (SRH) information and services (Tebb & Brindis, 2022).

Prior studies have documented barriers and facilitators to accessing SRH, highlighting the influences that interpersonal perceptions play on the teenager’s own SRH decisions (Kamke et al., 2022). In a recent study that sheds lights on barriers to accessing SRH care identified by youth, parental disapproval, stigma, embarrassment, and confidentiality concerns were highlighted. In contrast, facilitators included peer and family support, education and awareness that services are provided in a confidential manner, and accessibility, including location and convenient clinic hours of operation (Decker et al., 2021; Teagle & Brindis, 1998). Other research has noted the role of existing cultural and religious norms, (reflecting both local customs, as well as those of recent immigrants), and how they add structural and contextual barriers to accessing SRH education and care (Chuang et al., 2012). Adolescent immigrants face further challenges and barriers related to their immigrant status, such as concerns regarding seeking care as it might lead to deportation (their own or other family members), varying public charge requirements or misinformation regarding their eligibility to access care, and racial and structural discrimination (Cristancho et al., 2008). These factors combined contribute to current disparities in TP rates, reflecting truncated opportunities for youth to remain pregnancy free during their formative years (CDC, 2021).

The Socio-Ecological model provides a useful framework for understanding the complexity of factors at multiple levels that influence perceptions surrounding TP. This study’s focus is on key community stakeholders as they hold important roles that intersect several levels within the Socio-Ecological framework. They include actors that shape and influence the environment and context at each of the model’s levels. For example, healthcare providers may mostly operate at the institutional level, but as designated community leaders, they also represent the first line of defense in implementing macro issues, such as laws, policies, and protocols that shape whether teens can easily access confidential care. Similarly, school stakeholders may be most influential in implementing policies within the school setting that mirror cultural and policy values, for example, whether a pregnant teenager remains in school both during her pregnancy and as a mother, or whether school educators follow, or do not follow, state mandates to offer comprehensive sex education. By considering different levels of influence, as well as individual’s own beliefs and values, greater insights can be gained to further elucidate factors involved in sexual teenage decision making.

### Changing Rural Demographics Shaping the Issue of Teenage Pregnancy

Disparities are confounded with changing demographics in the U.S., especially in rural areas. Attracted by a growing meatpacking industry, first generation immigrants, predominantly from Mexico and either Spanish- or Indigenous-speaking populations, settled in the Midwest over the past 3 decades (Housing Assistance Council, 2012; Cristancho et al., 2008). Recent demographic changes are reflected in wide ethnic and racial group variations living in traditionally White-only geographic areas. At the time of the data collection for this study, (2016–2017), 12% of the population in Kansas was Hispanic, while the national average was 18%. However, in specific counties, Hispanics represented 50% in Finney County, 55% in Ford, and 61% in Seward (Pew Research Center, n.d.). Teen birth rates in these counties were higher than the Kansas state rates of 21 per 1000 15- to 19-year-olds: respectively, 71, 70, and 83 per 1,000 15- to 19-year-olds (County Health Rankings & Roadmaps, n.d.).

### The Unique Aspects of Providing Teenage Pregnancy Prevention Interventions in Rural Communities

The U.S. Department of Health and Human Services has identified multiple successful evidence-based interventions for TP prevention (CDC, 2020), including several focused on diverse ethnic and racial communities (CDC, 2018). However, evidence-based interventions implemented in rural settings are rare, limited in scope, and do not address the unique needs of Latino adolescents and families living in immigrant, low-income, low-resourced rural communities (Goesling et al., 2018). Furthermore, most interventions have not fully incorporated a Socio-Ecological approach into programs implemented, with most focused on shaping individual behavior, rather than also focusing on proximal and external influencing factors, despite their important impact on teen’s interpersonal interactions, available options, and their consequent behaviors (Brindis & Moore, 2014).

For new interventions to succeed among Latino vs Latina teens living in such communities, it is important to address key stakeholders’ perceptions of TP that are embedded in the intersection of rurality with race, ethnicity, social norms, and immigration status. To develop effective interventions, contextual factors, such as demographic trends, must also be addressed. Although a few studies have investigated TP perceptions in rural areas in the U.S. (Dickson et al., 2020; Yarger et al., 2017), there is limited literature that specifically describes key stakeholders’ perceptions regarding TP in emerging, immigrant, rural Latino communities.

The gap in research on key-stakeholders’ perceptions limits the availability of culturally congruent TP curriculums and impacts societal norms and access to reproductive health education and healthcare for Latino adolescents. Furthermore, their perceptions pertaining to TP are particularly relevant given contextual changes in national and local immigration enforcement policies over the past few years, the controversial nature of immigration to the U.S., and its negative impact on the overall health of Immigrant Latinos living in the U.S. (Rhodes et al., 2015). Using brief demographic surveys and semi-structured interviews reflecting the domains of the Socio-Ecological framework, the objective of this formative study was to fill existing data gaps regarding the perceptions and beliefs among key stakeholders representing three emerging immigrant rural Latino communities in southwest Kansas and implications for future TP efforts.

## Methods

### Study Design and Sample

Incorporating the Socio-Ecological model’s four bidirectional levels of influence on teenagers’ behaviors (macro, community, institutional, and interpersonal levels), we recruited key stakeholders to ensure representation across all levels. Specifically, a purposive sample of key stakeholders was drawn from three rural counties in southwestern Kansas (Finney, Ford, and Seward counties) to participate in this formative research. Recruitment was based upon five distinct roles they played in the community to help provide further insights regarding their roles in shaping adolescents’ environments. Prospective participants were contacted at their work sites, including healthcare waiting rooms and community-based agencies, by a trained bilingual community liaison and members of the research team to ensure a diverse sample of community health workers (CHWs), healthcare professionals (HCPs), public health officials, school health officials, and community members.

### Procedures

Criteria for inclusion included if potential participants belonged to any of the 5 aforementioned groups and spoke either English or Spanish. Interested participants provided verbal consent and invited to complete a brief, confidential demographic survey in advance of a semi-structured interview. Research instruments could be completed either in English or Spanish at a convenient time for the respondent. Survey questions collected respondents’ background information, as well as information on their job title. Interview guides included questions on participants’ beliefs regarding the role of culture in perceptions regarding TP and SRH behaviors, contraception use, and obstacles to TP prevention. All participants received compensation of a $50 gift card and were offered light refreshments. Both the University of Kansas Medical Center and Children’s Mercy Hospital’s Institutional Review Boards approved this study.

All study procedures were conducted from November 2016 to May 2017. A sample of convenience was recruited and interviews were conducted until reaching data saturation points, thus, interviews were conducted until no additional themes were being identified from which the team could develop additional data response categories (Strauss & Anselm, 2017). Only a few participants, equally from each setting and level of influence, turned down the opportunity to participate.

### Measures

*Survey questions:* included participant demographics such as age, gender, level of education, religion, race and ethnicity, and nationality. In addition, participants reported on their job titles and where they worked. This background information was used to analyze the interview themes in the context of the individual’s level of influence.

*Semi-structured interviews:* included assessment of personal and cultural beliefs and attitudes towards TP, and overall perception of sexual health and reproductive health use among teens, as well as obstacles to TP prevention, including access to contraception (see Table 1).

**Table 1:** Questions utilized in interviews to query teenage pregnancy prevention perceptions and beliefs

**Beliefs and attitudes**	**Questions utilized in interview**
**Participants’ beliefs regarding teenagers’ health, pregnancy, and contraceptive use**	• Community members were asked: – What comes to your mind when you think about sexual health, pregnancy, and contraceptive use by teenagers? – What resources do you have available to find more information about sexual health, teen pregnancy, and contraception? – What resources do you have available for any question that may exceed your comfort level? – Can you think of someone you could refer teens to talk more about these topics? • Healthcare personnel were asked*:* – What services do pregnant teens need? What services tend to be more helpful and available? – What services do you find more difficult to support? – What is your opinion about different contraceptive methods for adolescents? What type of contraception do you feel most comfortable providing to adolescents?
**Participants’ beliefs regarding potential obstacles to Teenage Pregnancy (TP) prevention**	• All participants were asked: – Tell me about individuals or groups that would/would not support you in discussing sexual health issues, teen pregnancy, and contraception with teenagers? – What do you think is the role of religion as it relates to teenagers’ sexual health, pregnancy, and contraception?
**Role of culture in perceptions regarding teen Sexual and Reproductive Health (SRH) behaviors**	• All participants were asked: – How do Latino families and teenagers feel about sexual behavior, teen pregnancy, and contraception compared to other racial/ethnic groups? – What is specific or unique to Latino families dealing with reproductive health services, teen pregnancy, and contraception? – How does the Latino culture influence these topics, for example, the way Latinos think about teenage pregnancy? – What are the main barriers Latino families encounter in accessing reproductive health services for their teens? How do health service referrals change for non-US citizens? – What do you think is the role of religion among the Latino community regarding teenage sexual behavior, teenage pregnancy, and reproductive healthcare?

### Data Analysis

Demographic survey data were manually entered by research staff into Research Electronic Data Capture (REDCap) (Harris et al., 2009) and quantitative survey data were analyzed using REDCap version 6.11.5. Descriptive statistics were reported for each survey item pertaining to demographic characteristics and job categories and roles.

Qualitative data was analyzed using Dedoose qualitative software (Salmona et al., 2019). Thematic categories were organized according to how they addressed TP perceptions—both from the perspective of what opinions the stakeholders held about teenagers themselves, as well as other key stakeholders in the lives of teenagers, including parents and providers. Trained bilingual research staff transcribed the audiotaped interviews.

Two trained bilingual research staff members (RB, AG) followed grounded theory principles and initially examined interview transcripts line-by-line to assign “open codes,” capturing concepts relevant to the research question in that data segment. Through a systematic and iterative process, the team then drew a connection between codes (“axial coding”), followed by selective coding (selecting one central category that connected all the codes from the analysis), and developed a codebook that reflected main themes and subthemes which helped to generate nuanced findings, verifying reliability (Strauss & Anselm, 2017). Discrepancies in codes were identified and resolved through consensus within the coding team, reaching 94% agreement. Importantly, we followed a socio-ecological theoretical framework during our coding, and as themes emerged, we described them within the levels of influence across the framework. We reviewed how roles and positions within the framework also shaped the adolescents’ environments.

## Results

### Sample Description

A convenience sample of 48 participants was enrolled and completed the demographic surveys followed by the interviews (Table 2). Respondents included 5 public health department staff (PHD), 8 community health workers (CHWs) (teen parent educators, pregnancy care center staff, community health coalition staff members), 8 healthcare providers (HCPs) (2 medical doctors, 2 nurse practitioners, 2 registered nurses, 2 certified nursing assistants), 9 community members (1 waiter, 1 janitor, 4 community leaders, 2 public officials including the county commissioner, 1 college student), and 18 high school/college administrators (4 counselors, 5 teachers, 1 college advisor, 4 administrative assistants, 2 federal program staff, 2 parent coordinators/advisor). The mean age of participants was 43 years (SD = 15.5). Most of the participants were female (85%; *N* = 41) and had attained at least some college education (38%; *N* = 18). Most participants were Christian/Catholic (72%; *N* = 33). Half of the study population were Latino/a (50%; *N* = 23) with more than half of the participants (63%; *N* = 29) born in the U.S. Of those 19 participants born elsewhere, most (80%; *N* = 15) were born in Mexico (Table 2).

**Table 2:** Selected participant demographics (*N* = 48**)**

	***N* (%)**
Mean age, years (SD)	43 (15.5)
Female	41 (85)
Role in community	
Healthcare provider	8 (17)
Public Health Department staff	5 (10)
Community health worker	8 (17)
Community member	9 (19)
School staff	18 (37)
Religion	
Catholic/Christian	33 (72)
Protestant	7 (15)
Other	6 (13)
Education	
High school or less	10 (21)
Some college	8 (17)
Associate or bachelor’s degree	14 (29)
Advanced degree	16 (33)
Latino/a	23 (50)
Born	
In the US	29 (63)
In Mexico	15 (33)

### Interview Themes

Grounded theory analysis revealed 3 major themes, and 9 subthemes, with perspectives that varied by the different roles that the key-stakeholders played in their community, as well as the level of influence in which they operated.

*Theme 1: Teenage Pregnancy (TP) as a cultural norm:* For HCPs, PHD officials, and CHWs, TP was not unusual, nor “unexpected.”

Subtheme: TP is a common occurrence across the community. Most study participants shared that either themselves, a young family member, or friend had become pregnant during their teen years.

But I think it’s just the need to feel accepted. In a lot of cultures, mine included, my grandma had babies and was married when she was 16 years old. So, it’s not that uncommon for those families; that’s what they’ve seen, that’s what they’ve known and for a lot of them it’s worked tremendously well. It’s not always the case obviously; but they’ve seen those examples, so they think why not me. You know why it can’t work for me and my boyfriend; we’ve been… you know we love each other; we’re going to stay together forever, whatever… School official.

Subtheme: TP provides meaningful roles. TP was associated with an act of seeking love, attention, or connectedness with partners.

I think that our population is so busy, and our teenage girls are- trying so hard to establish connections with someone and they’re trying to get these boys to love them; and they think it’s going to be forever, and they have sex with them; they don’t protect themselves, they end up with the babies. Um… I think they think it’s the love is going to last forever. School official, counselor.

Subtheme: TP and lack of perceived opportunities. School officials added they thought teens were “not aware of their own potential” and becoming pregnant was related to the teenagers’ perceptions that they “lack opportunities” or secondarily, to “boredom.”

I think it’s… to me I think it’s really important, I think that sometimes these girls don’t realize their own potential. And I think in the smaller communities everybody is having babies. And so unfortunately culturally it’s the norm sometimes… School official.

They’re bored… they want to be accepted. I think it’s the same way, the same reason kids get in gangs. They want to belong to something and they… you know, I wished I knew. I think very little of it has to do with sex. Public Health staff.

Subtheme: Cultural responses to TP. Overall, immigrant parents, mostly from Mexico, did not expect sexual intercourse to happen before marriage, especially for girls. In fact, parental response to the disclosure of a TP in their families was initially treated as a disappointment, followed by welcoming the new life and providing support as part of a cultural commitment to family, even under undesired circumstances.

Well obviously, like any other Latino family… the initial reaction is anger, then obviously the planning to assist her to carry a healthy pregnancy… and help with the baby and obviously evaluate the state of relationship with the boyfriend or the individual or couple and ... just assist so she has a healthy pregnancy. Translated, Community member.

Subtheme: Pregnant teens support programs vs. TP prevention programs. Participants that worked in the community (PHD staff and CHWs) did not know of any local programs for TP prevention, but instead, mainly reported programs to support teens who were either pregnant or already parents. One such program was offered by the public health department and included a caseworker paying visits to the new mother’s home bringing resources like car seats, diapers, baby food among other economic resources for the new parent. Another program offered a meal and “points” for attending a school program for new teen mothers, while a third model available in one town was a small non-profit, traditionally supported by local donations, that mainly provided pregnancy tests and pro-life support to pregnant teen parents. Staff did not provide information on contraception or abortion options but mainly provided information about abstinence and pregnancy tests. They emphasized support for “keeping the baby” and provided diapers, baby food, and other resources for new moms.

Once she delivers, if they want to get involved um…Catholic social service has a teen mom’s group, which is a fabulous group. She does it in the schools, if there’s enough that can attend, but she also does it on Wednesday nights and I think it goes up until 23 girls, but um- she just has different members from the community that pair up and mentor the teen girls. So again, that’s just that extra adult in their life to make sure they’re getting what they need. And then she brings in different resources too. And each time they come to the meeting or whatever, they can earn teen mom bucks. They have a little store there and they can buy diapers, formula, clothes, whatever they need for the baby. It’s just a meal- they share a meal together that’s brought in by the volunteers. They bring in, like I said, different resources from the community. Again, they continue to talk about their education, taking interest surveys; to you know ‘what do you want to do for career and how do we help you get there’ and stuff like that… School official.

*Theme 2: Obstacles to accessing Sex Reproductive Health (SRH) education and confidential healthcare*: Overall sex education was regarded as taboo and most parents did not feel comfortable opening these conversations with their children at home.

Subtheme: Obstacles to teaching sex education in the community. Parents voiced a concern of sounding permissive if holding or allowing these conversations to occur, including permitting sex education in school. Parents also felt sex education was provided “too early” in schools. CHWs echoed the perspective of community members in stating that sex-related conversations were not welcomed nor openly held in households, particularly before marriage.

I think the kids take our guidance, but it’s the parents that are sometimes more set in their ways and don’t want those conversations. And they tell us a lot of times that they don’t want the school having those conversation because they’re going to have those conversations with their kids, but most of them don’t…. School Official.

This topic is still taboo. And here in school there is not much we can do. I know some other schools offer condoms … to the kids. Not here. It’s not forbidden but does not look good. The thought of most, and actually mine included, is that if they have condoms in their pockets, they are going to want to use it (translated)… CHW.

In contrast, HCP had previously worked with schools to provide sex education, but these programs were mostly cancelled once found to be “too comprehensive” and thus, education was currently limited to abstinence only. HCPs also expressed doubt that this approach would prepare students properly. Thus, health education mostly covered “PE and health.” Offering comprehensive sex education curricula clearly faced resistance among students’ parents and obstacles in being approved at the school-system level. These key stakeholders’ contrasting opinions are highlighted in Table 3.

**Table 3:** Contrasting primary themes as reported within and across different groups of key stakeholders

**Main themes**	**Health care provider**	**Public Health Department**	**Community Health Worker**	**School staff**	**Community members**
**1-Teen Pregnancy (TP) treated as a cultural norm** *- Pregnant teens support programs vs. TP prevention programs*	+	+	+	+	+
**2- Support for comprehensive sex education and Sexual Reproductive Healthcare (SRH)** *-Obstacles to accessing Sex education and SRH healthcare*	+	+	±	+	±
**3- Strategies to support teen pregnancy (TP) prevention** *- Open the conversation with parents first* *- Utilize ongoing family venues to implement educational workshops*	+	+	+	+	+

+ = this group agreed with statement; – = this group did not agree with statement; –/+ = this group had members that agreed with statement and others that did not

Subtheme: Obstacles to Sexual Reproductive Healthcare. HCPs readily reported that overall lack of being able to assure confidentiality for SRH services represented a strong barrier to teenagers’ accessing care. They noted that the already limited gynecological or SRH services were reported to be underused due to a lack of knowledge that these services even existed, but more frequently, due to the perception that parental consent was required to access these services. HCPs (school nurses), PHD, and school staff stressed the priority of offering additional SRH resources and services, but also acknowledged how this step would not be possible without fully engaging additional parental support.

Other than just going to their family doctor, but that’s up to their family doctor if they’re bound to tell their parents and usually by the time with insurance- I mean all that goes through insurance; the parents are going to find out anyways. So, I would say from the counselor point of view, and for students that are coming to us and they’re thinking about that, we always encourage at least trying to have that conversation with their parents. Make informed decisions with their parents. School official.

Another important concern was that the lack of free or reduced-cost healthcare, as well as lack of health insurance coverage or government support, such as the Title X Family Planning Program, needed for reimbursement, further limited access to available services. Health department officials voiced that for services that were available to uninsured patients, patient concerns centered on lack of “required documentation,” given their immigrant status, and fears of deportation should they attempt to seek care.

So, there’s times where I have to reassure them and tell them, you know I’m not here for that report due to immigrant status. So even just like their prenatal care, some of them are scared. They don’t want to go to the doctor. Because they’re afraid if they go, they’re going to get reported and they’re going to get in trouble. And I always tell them “You know you need to do your prenatal care, let’s not worry about all this other stuff. Take care of you and your baby.” And so, it helps a lot. And the health department is really good about you know the- we’ll do prenatal care. Public Health staff.

Another common concern for immigrants was the fear of discrimination when attempting to seek healthcare or other public services.

At the health department ... So basically, you don't get prenatal care till you're about to deliver... I really don't know, I really don't know…what makes a difference there as I tell you is the legal situation, if you don’t have documents, obviously it's a government service, that may require permanent residency or citizenship, and maybe you as an undocumented person or without documents will not be able to access those services. That's why I'm telling you, in our community it's a little harder for people who don't have documents. Translated, Community member.

School officials expressed that they were restricted in the healthcare-related services they could offer, since parental permission would be required for them to provide care, even including referrals to local agencies to access services. In contrast, parents saw that allowing SRH care would be seen as permissive, which was echoed by CHWs.

There was this girl I talked to, she was in high school, and wanted to know if I could take her to get birth control, and I told her I could not, but could tell her where to go, thou could not go with her. She was very scared of going and having to use her insurance, then having her parents find out. So, there’s no place that offers confidential services, there’s no way their parents won’t find out. Translated, School official.

These key stakeholders’ contrasting opinions are highlighted in Table 3. As noted, not all categories of stakeholders had consistent responses, depending upon theme. Key stakeholders who identified as Latino were more hesitant to support comprehensive sex education and SRH.

*Theme 3: Strategies to support teen pregnancy (TP) prevention:* Participants suggested specific strategies to support TP, which were consistent regardless of the types of groups they represented.

Subtheme: Open the conversation with parents first. There was an overall agreement that conversations on SRH topics “need to start at home,” highlighting the importance of having the whole family be “on the same page.” This statement was supported by community members and CHWs, who added there was a need to educate parents first, and bring educational programs home, to be able to positively engage parents. School officials, counselors, and school nurses suggested that without educating parents first, that it would be difficult to have teens access SRH education and services. They emphasized the importance of stepping away from a taboo approach to these topics, and instead, engaging parents so that they feel more comfortable having an open conversation with their children.

Parents need education on how to talk to their, their, young kids, so have a parent night, you know. And talk to them about how they need to open up and not just, um… “we need to talk about that later,” you know. Be able to help parents learn the language, to open up, and open-ended conversations and their kids. So not only at school or, with the young teens, help parents be able to communicate better. HCP.

Subtheme: Utilize ongoing family venues to implement educational workshops.

HWs suggested that SRH-related education had to start with parents and added that these programs could build on already ongoing activities where the whole family participates. Some of these family activities occur in community centers, churches, and schools, so building on that community trust seemed a good place to start. Participants did acknowledge that there would be a need for initial sessions just for the parents, while their children would be involved in other activities.

But I think that would be a way to do it, via workshops and promote them to those who want to go. Um, to allow parents to attend too, even workshops that, like, help the parents communicate with their children on the subject. Because, ignoring it doesn't work. School official.

Overall recommendations to decrease the occurrence of TP were offered by all key stakeholders across all groups. However, they did not offer the content specificity needed for program development and implementation, whether the programs were directed at teenagers or their parents.

## Discussion

Adopting a Socio-Ecological framework, this study is among the first to describe community key stakeholders’ perspectives and their own held beliefs about TP prevention in emerging immigrant Latino rural communities. Results can help us understand obstacles and facilitators for developing future TP prevention programming. As shown by these results, adolescent behaviors are shaped by many contextual factors and stakeholders who function directly or indirectly across the multiple layers that influence adolescent behavior, including the macro (policies, resource allocation and teen health priorities and program development), community (neighborhoods and community organizations’ support in terms of resources and program dissemination), institutional (school funding, implementation of comprehensive sex education), interpersonal (family knowledge of sex education and comfort in sharing values), and individual level (teenager’s own knowledge and behaviors).

### Macro, Community, Institutional, Interpersonal, and Individual Levels

At the Macro-level, stakeholders, such as healthcare providers, who often are called upon to be “first line responders” in implementing broader government policies, such as funding and implementation of confidential reproductive healthcare services, noted the lack of policy consistency in the environments in which teenagers need to navigate their transitions into adulthood. For example, while formal federal policy would allow teenagers’ access to confidential reproductive healthcare, local informal clinic policies and local norms makes such access far more constrained. Additional layers of complexity are shaped by stakeholders who work in rural environments with Latino immigrants, where politically sensitive fears of deportation and fear of public charge policies would preclude adolescents and their families from seeking any healthcare where expenses would be accumulated. Overall, particularly in isolated communities, local norms may be more compelling than formal policy “on the books.”

Across all stakeholders working in these rural Latino communities, there was wide acceptance and normalcy assigned to the prevalence of TP. Prior studies have also described intergenerational childbearing patterns and cultural and religious framing of childbirth within Latino communities (Dickson et al., 2020; Yarger et al., 2017). Our study highlights how these cultural norms are somewhat more acceptable in rural, emerging immigrant communities, where personal as well as professional experience, as well as ambivalence regarding TP, was frequent, given the perceived lack of viable alternatives for adolescents by both the adolescents and some of the adults.

At the community level, many of the stakeholders’ held ambivalent beliefs regarding their ability to influence and shape the environments in which they work. Despite the high incidence of TP, parents were perceived to be resistant to openly discussing sexual health topics as it was seen as condoning pre-marital sex, counter to cultural beliefs. Furthermore, many stakeholders themselves often shared the same normative belief system and felt that they would not have sufficient influence to change parental beliefs. Similar trends have been described in the literature within Latino families, where traditional values lead to limited discussions on topics related to sex, particularly for women, and especially before marriage (Guillamo-Ramos et al., 2006). These patterns are perpetuated within rural settings as documented in other studies, where HCPs also noted that “in the Latino culture, people do not talk openly about birth control, sexually transmitted diseases, or sexuality.” (Branch et al., 2010).

At the institutional level, results suggest that there was clear ambivalence when it came to supporting sex education among different stakeholders. While HCP, PHD, and schools supported such programs in schools, lack of consensus persisted among CHWs and community members, which resulted in less likelihood of having such curricula available to students. The professional HCP and PHD participants aligned with research that has documented that lack of comprehensive sex education is strongly associated with worse reproductive health outcomes for adolescents (Santelli et al., 2006). Our results showed that participants who identified as Latino/a held more conservative beliefs and were less prone to support comprehensive sexual education and SRH services regardless of their stakeholder positions (not shown in tables). Our findings contrast with the findings of a recent meta-analysis (between 2000 and 2016) that documented high levels of positive adult and parent attitudes toward sex education resulting in more welcoming attitudes, being more open to having SRH discussions in school, and making such education readily available (Szucs et al., 2022). Of note, this meta-analysis did not analyze whether rural, low income, and Latino immigrant parents were included in any of the studies. These results point to the need for smaller geographic-related studies to help differentiate how demographics, geography, culture, and religious beliefs, as well as socio-economic status among different key stakeholders, may result in significantly different results than those documented in broader samples.

At the interpersonal level, this study adds to the literature by pointing to the role that ambivalence and lack of consistency among key adults, including supporting access to SRH care for teens, plays a key role in shaping options available for teenagers in the very institutions that are supposed to provide this type of support. While HCP, PHD, and school staff supported SRH, lack of consensus persisted among CHWs and community members. This lack of consistency among adults contributed to a more challenging environment for teenagers, who have less cognitive ability to reconcile different points of view with those that they interact with frequently, particularly, as they are more likely to be directly supervised by their parents and other community adults (Miller, 1989). This phenomenon has been described in the literature before, citing parental disapproval as an important obstacle for Latino teenagers’ access to SRH both in rural and urban areas (Decker et al., 2021). This study underscores the intersection of individual characteristics and roles of additional, influential adults surrounding teenagers and their level of support or non-support for a range of health topics, skills, and competencies needed to successfully navigate their adolescence with the information they need to avoid facing an unintended pregnancy. The inconsistencies further constrict adolescents’ efforts to pursue protective health behaviors, as well as their ability to act independently when they are unable to reconcile diverse explicitly and non-explicitly stated cultural and structural messages. Such challenges have become even more profound with the Supreme Court Dobbs’s decision which makes access to abortion access, and possibly contraceptives, even more difficult in a growing number of primarily rural, states (Kaufman et al., 2022).

At the individual level, personal issues were noted by respondents, including the ability of adolescents to have the skills they need to deal with geographical isolation and limited knowledge regarding how to access health services on their own. Furthermore, reproductive SRH requires a level of confidentiality, particularly for adolescents, that is difficult to ensure in small towns, especially when false narratives regarding the need for parental consent to access birth control are held by those adults that adolescents rely upon for information (Chuang et al., 2012). Our study further dissects the extent to which these limiting factors are further exacerbated among emerging Latino immigrants, as these communities often lack the social capital, support networks, and familiarity navigating the complexities of the U.S. healthcare system (Topmiller et al., 2017). Specifically, our participants brought up concerns regarding immigration status which limits access to health insurance and related SRH care for new immigrants. Importantly, discrimination was also a theme among our participants as an obstacle to healthcare access. Of note, data collection for this study occurred during a politically heated environment regarding immigration policy that increased the health burden on immigrants (Rhodes et al., 2015). It is reasonable to suspect that the challenges we identified may be of even greater concern given the ongoing COVID-19 pandemic, controversy regarding immigration status, and a post-Dobbs environment that is chilling access to reproductive health services for adolescents and adults alike (Kaufman et al., 2022).

### Next Steps

Our participants identified potential solutions to current obstacles faced by teenagers living in these isolated communities, when trying to access SRH education and services. School personnel and community members agreed that sex education programs tailored for parents were needed as a first step towards greater acceptance. Our findings are aligned with other TP prevention efforts for Latino immigrants in rural Arkansas, where parents voiced suggestions to improve the proposed sex education programs, and gravitated when the entire family was invited to participate, not just their children (Murphy-Erby et al., 2013). Overall, our study adds suggestions for successful TP prevention programs in rural Latino immigrant communities, to specifically target the immigrant family as a unit, incorporating them in ongoing efforts and familiar, trusted venues, highlighting the family centeredness or “familismo” (e.g., a cultural norm that involves individuals’ strong attachment and solidarity to their nuclear and extended families) (Brindis & Moore 2014). However, these suggestions lack concrete details that could inform the development and implementation of TP prevention programs, such as specific content and logistics for approaching a taboo topic in a community with strong cultural norms and religious beliefs.

These study findings suggest that conflicting community stakeholders and contexts can result in ambivalent messages for teenagers to interpret when it comes to expectations surrounding TP. These conflicts reflect the intersection of multiple challenges, including conservative perspectives on sexuality and family formation, constricting immigration policies, and rural settings with inadequate resources to assure confidential care. To overcome these obstacles will require acknowledging cultural norms and constraints and the development of additional strategies that both respond to strongly held cultural norms and beliefs, such as family-focused approaches offered in trusted settings, while also opening new approaches for protecting young people.

## Limitations

There is the possibility of a selection bias in our sample since a convenience sample may end up excluding other community voices from the results. The voluntary nature of participants means that people who are inclined to know about the subject or are more positive about a topic may be more likely to participate. To offset this bias, we pursued a variety of participants in diverse settings that captured contrasting viewpoints, including those who supported comprehensive sexual education and SRH services and those who opposed them. These contrasting viewpoints are highlighted in Table 3 and reflect the ambivalence expressed regarding TP, which can influence the level of support towards SRH education and care.

Another limitation includes the reliance on qualitative analysis, which could be subject to potential bias among the investigators. However, the range of findings across different stakeholders and their insights and nuanced understanding of concepts, opinions, and experiences regarding TP prevention in groups that have been historically marginalized offer these results face validity. Despite the study’s data being collected from 2016 to 2017, recently resurfaced national and local legal bans on access to SRH and the impact of the Dobbs decision on state policies have led to perpetuation of restriction of adolescents’ access to SRH care (Kaufman et al., 2022).

Furthermore, current ongoing debates and increasing controversies regarding the teaching of sex education curriculum in classrooms continue to make this research relevant (WUSF Public Media, 2023). Additionally, to our knowledge, this study continues to be the only one that describes the impact of the multi-layered “adult” environments in which adolescents are surrounded by, which often may not be consistent, leading to an inconsistent and potentially unsupportive environment in which to make teen’s decisions.

## Conclusions and Implications

Further qualitative and quantitative studies are needed to help develop solutions that directly impact the provision of TP prevention education and SRH care among rural, immigrant Latino youth, including strategies that specifically address cultural biases and norms. Furthermore, strategies that are both culturally specific, while also helping to educate parents on how to best protect their children from an unintended TP, are needed.

These findings suggest inconsistent perspectives regarding support for SRH education and access to care and lack of consensus among key stakeholders can lead to negative consequences for adolescents’ ability to make health-promoting choices. Conflicting messages can leave cognitively inexperienced teenagers with fewer skills they need to reconcile divergent and unclear messages. On the potential positive side, if perspectives can eventually be reconciled, it provides adolescents with the possibility of receiving education once parents become more knowledgeable about the potential risks if their children do not have the knowledge, nor skills they need to avoid an unintended and mistimed pregnancy.

Taken collectively, these findings framed within the Socio-Ecological framework reflect the intersection of rurality with culture and immigration that Latino teenagers face in emerging immigrant rural communities, as well as the multiple stakeholders in their environments which influence the options teenagers have available. Identifying and disaggregating perceptions of influential stakeholders at each level can help inform key stakeholders, such as parents, communities, educators, policymakers, and others in efforts to develop culturally responsive and community-engaged TP prevention programs. Awareness of these perspectives can help better address the unique social, economic, and structural determinants of health for adolescents in emerging rural Latino communities. This is particularly relevant given changes in national and local immigration enforcement policies over the past few years, the controversial nature of immigration to the U.S., and its negative impact on the overall health of Immigrant Latinos living in the U.S.

## Data Availability

N/A.

## Code Availability

N/A.
